# Aging and Visual Counting

**DOI:** 10.1371/journal.pone.0013434

**Published:** 2010-10-18

**Authors:** Roger W. Li, Manfred MacKeben, Sandy W. Chat, Maya Kumar, Charlie Ngo, Dennis M. Levi

**Affiliations:** 1 School of Optometry, University of California, Berkeley, California, United States of America; 2 Helen Wills Neuroscience Institute, University of California, Berkeley, California, United States of America; 3 Smith-Kettlewell Eye Research Institute, San Francisco, California, United States of America; University of Leuven, Belgium

## Abstract

**Background:**

Much previous work on how normal aging affects visual enumeration has been focused on the response time required to enumerate, with unlimited stimulus duration. There is a fundamental question, not yet addressed, of how many visual items the aging visual system can enumerate in a “single glance”, without the confounding influence of eye movements.

**Methodology/Principal Findings:**

We recruited 104 observers with normal vision across the age span (age 21–85). They were briefly (200 ms) presented with a number of well- separated black dots against a gray background on a monitor screen, and were asked to judge the number of dots. By limiting the stimulus presentation time, we can determine the maximum number of visual items an observer can correctly enumerate at a criterion level of performance (counting threshold, defined as the number of visual items at which ≈63% correct rate on a psychometric curve), without confounding by eye movements. Our findings reveal a 30% decrease in the mean counting threshold of the oldest group (age 61–85: ∼5 dots) when compared with the youngest groups (age 21–40: 7 dots). Surprisingly, despite decreased counting threshold, on average counting accuracy function (defined as the mean number of dots reported for each number tested) is largely unaffected by age, reflecting that the threshold loss can be primarily attributed to increased random errors. We further expanded this interesting finding to show that both young and old adults tend to over-count small numbers, but older observers over-count more.

**Conclusion/Significance:**

Here we show that age reduces the ability to correctly enumerate in a glance, but the accuracy (veridicality), on average, remains unchanged with advancing age. Control experiments indicate that the degraded performance cannot be explained by optical, retinal or other perceptual factors, but is cortical in origin.

## Introduction

Over a century ago, Jevons [1] addressed the question of how many objects the mind can “embrace at once”, by enumerating the number of beans that fell into a box, in a single glance. Since Jevons' remarkable study, much of the focus has been on his finding that one can apprehend up to 4 items in a glance without error, and that the number of errors increased in proportion to the number of beans. Largely ignored until recently is Jevons observation that, beyond 4, numerical enumeration even for large numbers of beans, is on average, quite veridical, the errors being about 0.12 times the number (i.e., it obeys Weber's Law).

Jevons finding, and subsequent studies involving response time measurement suggested that enumeration may be characterized by two distinct components [2]–[6]. (1) Subitizing [7]. When the number of items is small, i.e. fewer than 3 or 4 items, the process of enumeration is relatively automatic, effortless and error free (∼100% correct). Subitizing is very rapid, with each dot adding 50–100 ms to the response time, and is thought to be mediated by pre-attentive parallel processing limited by the available slots of working memory. (2) Counting. Once the numerosity is beyond the subitizing range, i.e. 4 items or more, the process of enumeration requires more effort and becomes more error-prone - involving cognitive processes (e.g., a shift of visual attention to search each dot spatially and to count serially), with each dot adding 300–400 ms, therefore counting has been hypothesized to be mediated by attentive serial processing. A more recent view is that the ability to apprehend numbers reflects a primary sensory attribute [8], [9], that is independent of density, possibly reflecting the responses of neurons in parietal cortex that are tuned to numbers (see [10] for review). Besides humans, other primate animal species, for example monkey, also demonstrate competent numerosity processing ability [11].

Visual counting task has shown to be useful in evaluating the integrity of the visual pathways. For example, strabismic amblyopes make errors, even with small numbers, and for larger numbers, markedly undercount (the number of dots reported is less than the number of dots displayed) the number of features [12]; this undercounting is suggested to reflect high-level cortical deficits in the number of features the amblyopic visual system can individuate. Visual enumeration has also been applied to evaluate other neurological conditions [13], [14].

Our interest is in the effect of normal ageing on visual enumeration. While many visual functions decline with increasing age, there is converging evidence showing that the speed of visual enumeration, both subitizing and counting (reaction/processing time per visual item) remains largely unchanged with advancing age [15]–[23], although a few of these studies reported mixed results, suggesting that subitizing range [20], subitizing speed [15], [17], [18] and counting speed [16] might deteriorate slightly. It is important to note that all these studies have focused on reaction time - i.e. how fast an observer can enumerate the number of items in a display and respond - rather than on the enumeration error of the counting process. Typically in these studies, an unlimited stimulus duration strategy was adopted: the stimulus remained on the screen until the observers recorded their responses, and they were allowed to make eye movements to search, and enumerate the dots during the test [22]–[24]. Not surprisingly, in this way enumeration error was reported to be very low, correct response rate always over 95% [15]–[23], thus showing age did not have an impact on the results. These findings basically reveal that when elderly observers are given enough observation time, they can perform as well as young observers. However, it is not yet clear how the normal aging process affects the apprehension of number in a glance.

Our study set out to examine the effect of normal aging on visual enumeration in brief displays. Instead of using unlimited stimulus duration as in the previous studies, our observers were presented stimuli for a very brief duration (200 ms). By limiting the stimulus presentation time, we can determine how many items an observer can enumerate, without confounding the experiment by eye movements. The methodology is identical to our earlier studies in examining counting accuracy in amblyopia [12], [25], and other studies in normal adults [26]. In the present study, we examined accuracy (directional enumeration error), variance (threshold) and speed of visual counting in observers over the lifespan, from 21 to 85.

In the elderly eye, there is reduced retinal illuminance (resulting from smaller pupil size, senile miosis) [27], reduced ocular transmittance (increased light absorption by the ocular media) [28], and increased light scatter [27]. In principle, these optical factors could degrade visual enumeration. To ensure that any age related changes were due to genuine neural changes specific to the visual enumeration process, we conducted a series of control experiments to eliminate these optical factors, and to evaluate other perceptual factors.

## Materials and Methods

### Observers

We recruited one hundred and four observers between 21 and 85 years of age with normal vision. For purposes of analysis we divided them into five age groups, about 20 in each group: 21–40, 41–50, 51–60 and 61–85 years (Table 1). All observers underwent a thorough eye examination. The maculae of all observers were assessed as normal; they had no drusen or abnormal pigment changes in an area of about one disc diameter around the macula. All observers had clear ocular media, as assessed by direct ophthalmoscopy, and were free of lens opacities in the natural pupil area. They had no manifest ocular diseases, nor did they have strabismus or amblyopia. All had normal or corrected-to-normal visual acuity (Snellen 20/20 or better) in both eyes. Viewing was binocular with full optical corrections; presbyopic observers were given an extra plus 0.25D lens to compensate for the testing distance (4 m) when necessary. The measurements took about 45 minutes. The task was self-paced, and observers were given breaks upon request.

**Table 1:** Characteristics of five age groups.

Age group(yrs)	Samplesize	Gender(M)	Gender(F)	Mean age(yrs)	SD(yrs)
21–30	20	9	11	22.6	2.6
31–40	18	10	8	34.0	2.9
41–50	20	10	10	45.0	3.2
51–60	21	11	10	55.3	3.3
61–85	25	10	15	68.8	6.6

### Ethics Statement

The experimental procedures were approved by the University Committee for the Protection of Human Subjects, and the research was conducted according to the principles expressed in the Declaration of Helsinki. The experiments were undertaken with the understanding and written consent of each participant.

### Visual Enumeration

**Figure 1:**
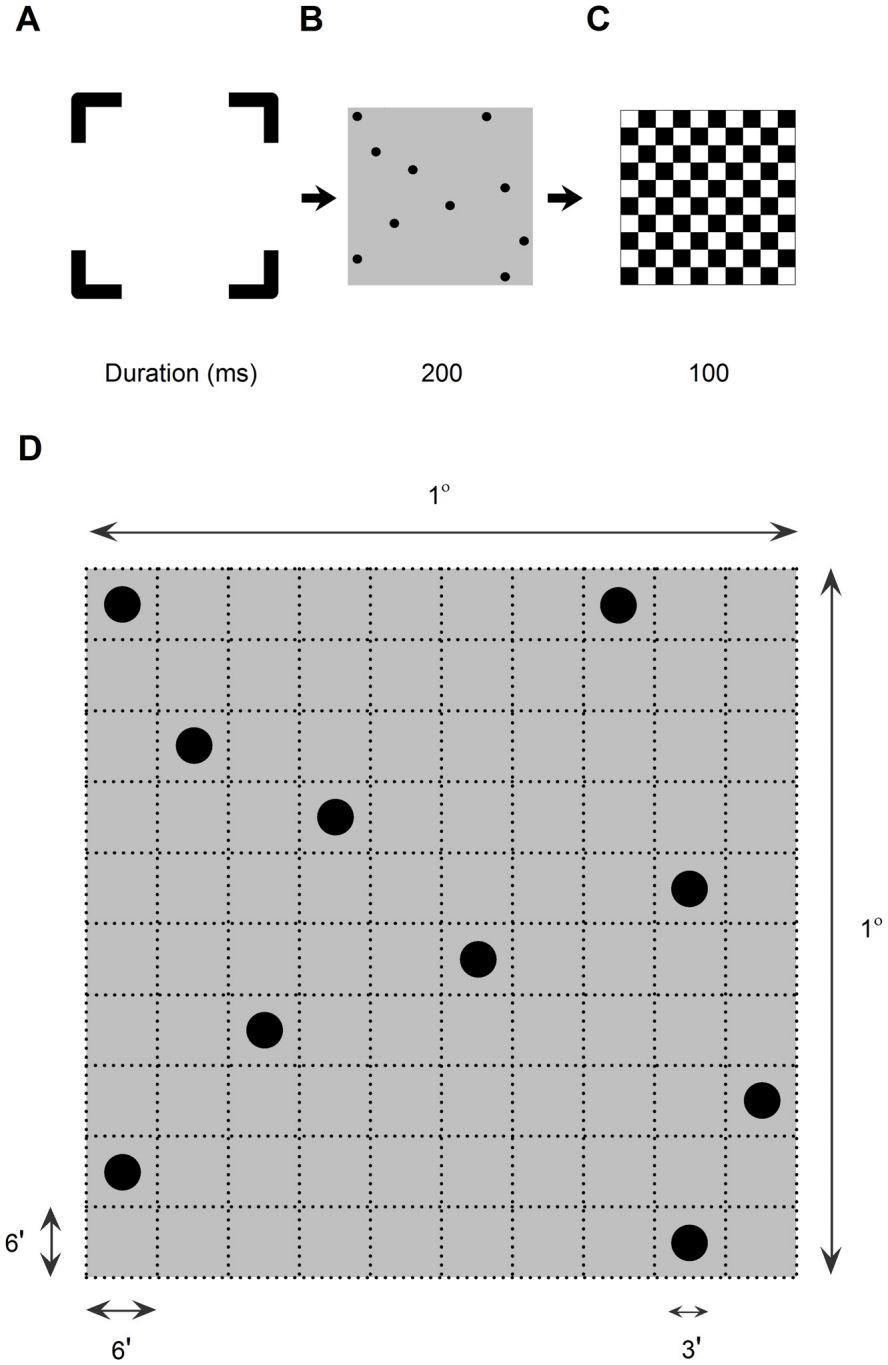
Visual stimuli. The stimulus sequence started with a fixation mark (a), and then a counting target for 200 ms (b), which was then followed by a black-and-white checkerboard mask for another 100 ms (c). Note that the fixation target was presented in a gray background, instead of a white background. (d) An example illustrating the design and physical dimensions of the dot stimulus. The task is to enumerate the number of dots (*N* = 1–10) in the display, and say the number into a microphone for the measurement of response latency.

A schematic diagram of the visual stimulus is illustrated in Figure 1. The stimuli were displayed on a 21 inch flat monitor screen (Sony F520) at 1800×1440 resolution and with a 90 Hz refresh rate. Each trial started with a “bracket” shaped fixation mark (Fig. 1A); indicating the upcoming stimulus location and area on the screen. A number (*N*) of highly visible black circular dots (0.5 cd/m2) was then displayed for 200 ms (Fig. 1B) against a gray background (42 cd/m2), with Weber contrast of 99%. *N* ranged from 1–10 dots; the dots were randomly positioned in 10×10 square cells (Fig. 1D). Each dot subtended 3 arcmin in diameter and was centered in its corresponding cell (6 arcmin ×6 arcmin); the entire dot stimulus field subtended 1° by 1° at a testing distance of 4 m. The distance between dots was at least two cells (edge-to-edge distance, ≥9 arcmin) to avoid resolution difficulties or crowding. The target stimulus was then followed by a checkerboard pattern for another 100 ms (Fig. 1C), which was used to mask any after images of the dot stimuli.

**Figure 2:**
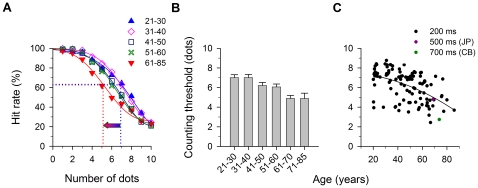
Counting threshold. (a) Mean hit rate as a function of the number of dots. A Weibull function was used to fit the data. The curves were gradually displaced to the left with advancing age. Dotted lines show the counting thresholds for two age groups: 21–30- and 61–85-year-old. (b) Mean counting thresholds and standard errors for different age groups. To better display the variation in counting threshold in older adults, the age group 61–85-yr was split into two groups here for visualization: 61–70- yr-old and 71–85-yr-old. (c) Threshold data for individual observers (n = 104) as a function of age. A second-order polynomial function was used to fit the data. Two older observers failed to perform the task for 200 ms, therefore the stimulus duration was increased to 500 ms (dark pink circle: JP) and 700 ms (green circle: CB).

Observers were asked to enumerate the number of dots as quickly and accurately as they could. Response latency was measured using the time it took to say the number into a microphone. Data acquisition of observers' voice responses was performed by an analog-to-digital converter (Measurement Computing Corporation, PCI-CTR05 board). No feedback was given with respect to observers' responses, and they were not given any information about the maximum number of dots to be displayed. Each block consisted of 100 trials, 10 trials for each *N*. To determine the precision of the judgment, we estimate a counting threshold. The thresholds reported for each observer were based on four blocks of measurement, i.e. a total of 400 trials. Prior to data collection, observers were given a practice session consisting of 100 trials. The response data of percent correct as a function of *N* was fitted with a Weibull psychometric function, and counting threshold was taken as the midway point between the upper and the lower free floating asymptotes as illustrated in Figure 2A (dotted lines; in this example, counting threshold refers to the number of dots at which ∼63% correct level was obtained). A similar threshold estimation strategy was adopted in our earlier studies [12], [25].

### Control Experiments

As evident in other aging studies [29], both optical and neural changes with age could affect visual performance. Since we are primarily interested in neural changes caused by aging, it is important to rule out any potential optical changes that might affect visual enumeration. A series of control experiments was conducted so as to consider various potential factors.

#### (1) Optical changes

Retinal illumination is reduced in the elderly eye, resulting from smaller pupil size and reduced ocular transmittance. The average pupil size is approximately 3 mm in older people (>60 yrs) compared to 5 mm in the youngest group (21–30 yrs) [30]. To eliminate these factors, we performed a separate experiment to investigate the role of the reduced retinal illumination in visual counting. To control for pupil size, we had five younger observers (20–40-yr-old) perform the same counting task with an artificial pupil of 3 mm, which was carefully centered on the observer's pupil and was placed about 1 cm in front of the cornea, over a neutral density (ND) filter. We used a 0.2 log unit filter (Kodak Wratten gelatin filter No. 96) to simulate the increased light absorption by the ocular media [31].

#### (2) Retinal (visual acuity) changes

Although our older observers had visual acuity of 20/20 or better, we cannot completely rule out changes in acuity, since visual acuity gradually decreases from 20/12.5-20/16 to 20/20 with increasing age [32]. To address the question of possible loss of acuity, the five younger observers (with acuity of ∼20/16) were tested again with optical blurring using plus lenses to reduce their acuity to 20/20. This control experiment was aimed at studying whether subtle retinal changes could lead to degraded performance.

#### (3) Practice effects

Perceptual learning is useful in improving visual functions [33], [34]. In a separate experiment, we asked whether practice could improve visual counting performance. Following practice, older adults might be able to perform as well as younger adults. Therefore, two elderly observers (KG, 67-yr-old; MA, 65-yr-old) with the lowest counting thresholds were selected to repeatedly practice the counting task (6 sessions in total; each with 400 trials), in order to evaluate the effect of practice.

#### (4) Stimulus duration

Performance might reflect age-related alternations in temporal integration time. To investigate this factor, we varied the stimulus duration in order to see whether longer stimulus duration could improve counting accuracy in three older observers (67–86-yr-old).

## Results

### Counting Thresholds

Figure 2A shows the mean hit rate (percent correct) as a function of the number of dots for each age group. The counting threshold reflects the maximum number of dots that can be correctly enumerated at a criterion performance level (≈63% correct), and was estimated by fitting a Weibull psychometric function to fit the data. The rightmost blue curve shows the data for the youngest age group. As reported by Jevons (who was his own observer, n = 1), for 3 dots or less, the hit rates were almost 100%, and decreased gradually to approximately 20% for 10 dots. With advancing age, the curves shift gradually to the left. The arrow in the figure indicates the difference in counting thresholds between the youngest and the oldest groups, equivalent to a change of about 30%. The mean counting thresholds were systematically decreased from 7.01±0.30 (21–30-yr-old) to 4.88±0.26 dots (61–85-yr-old) across different age groups.

To better display the variation in counting threshold Fig. 2B plots the thresholds versus age for each age group, and to clarify changes in the oldest adults (61–85) we split them into two sub-groups: 61–70- yr-old (n = 17) and 71–85-yr-old (n = 8). There was a statistically significant difference in the mean thresholds between the five age groups (ANOVA: *F*[4,99] = 9.278; *p*<0.0001). Post-hoc testing with the Tukey-Kramer test revealed significant differences between the 21–30 and 61–85-yr-old observers (*q* = 7.38; *p*<0.001), between the 31–40 and 61–85-yr-old observers (*q* = 7.17; *p*<0.001), between the 41–50 and 61–85-yr-old observers (*q* = 4.61; *p*<0.05), and between the 51–60 and 61–85-yr-old observers (*q* = 4.14; *p*<0.05), but there were no significant differences between the other age groups (*q* = 2.96 or less, *p*>0.05 in all cases). The differences among the standard deviations of all observer groups were not significant (Bartlett statistic = 0.14; *p* = 0.9977).

Figure 2C reports the individual thresholds across the age span, and shows the considerable individual variation at all age levels, with some of the youngest adults performing more poorly than some of the oldest. Note that two older observers failed to perform the task for 200 ms, the stimulus durations were thus increased to 500 ms (red circle: CB) and 700 ms (green circle: JP). A quadratic polynomial equation was used to fit the threshold data (*y* = −0.0005*x* ^2^−0.005*x*+7.51; *r* = 0.53).

### Counting Accuracy

**Figure 3:**
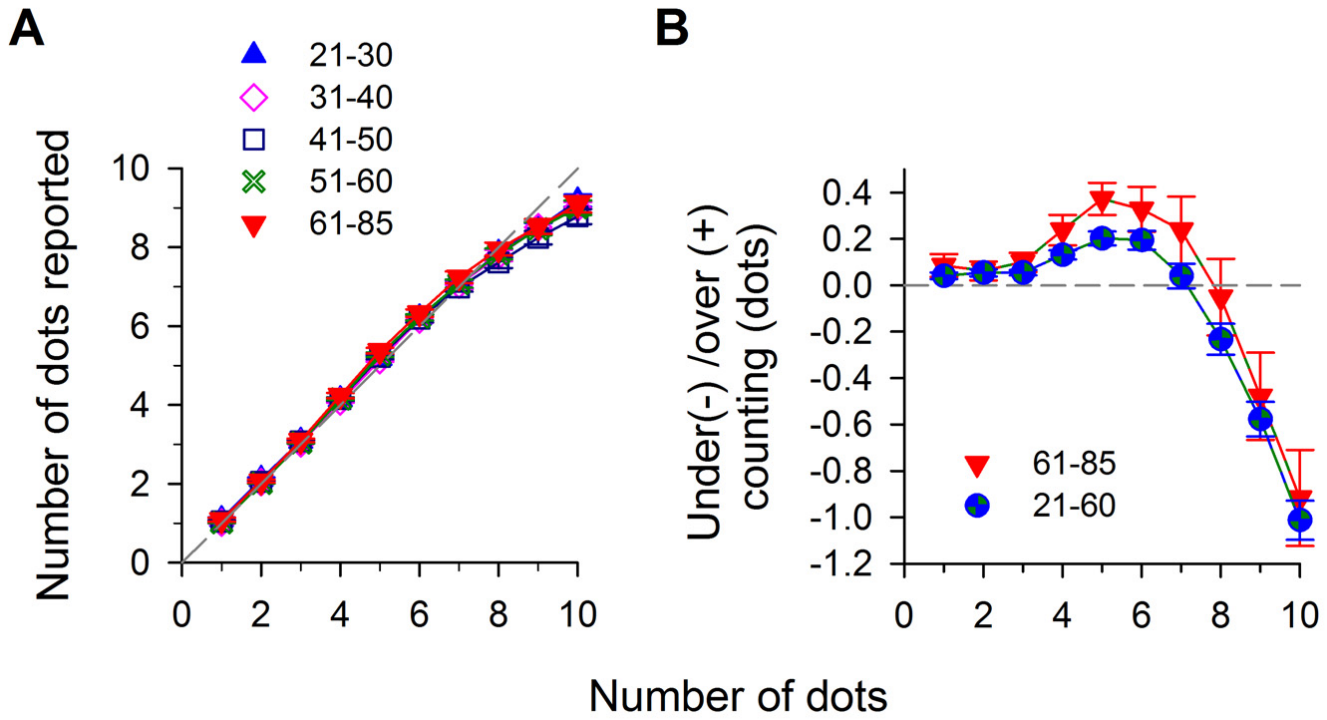
Counting accuracy. (a) Mean number of dots reported as a function of the number of dots displayed. In general, a very slight undercounting occurred when there were nine or more dots on the screen. (b) Undercountng/overcounting. The response accuracy data is replotted as signed derivation from the actual numerosity (number of dots reported - number of dots presented). Overcounting (+): more than the number of dots displayed. Undercounting (-): less than the number of dots displayed. Younger observers tend to overcount in the range of 4–6 dots and undercount thereafter, and older observers (red symbols) shows even more over-counting (relatively more positive in magnitude) when the numerosity is greater than 4.

If the reduced performance of the elderly were due to a limit in the number of items that the aging visual system can attend to and individuate, one might expect the elderly to systematically undercount the number of dots, much like strabismic amblyopes [12]. Surprisingly, despite the degraded counting thresholds in the older observers, their ability to count the number of dots was, on average, quite accurate (i.e. veridical - Fig. 3A). For all age groups, the mean number of dots reported was remarkably close to the number of dots displayed (1∶1 reference line), with a very slight undercounting for 9 or more dots (mean number of dots reported for all five age groups: 8.44±0.05); as for 10 dots presented, the mean number reported was 9.01±0.07. Thus, we can attribute the threshold loss primarily to increased random errors.

To look at this more closely, we plot the error of counting (taking into account the direction of the error – i.e., undercounting versus overcounting) versus number of dots (Fig. 3B). When viewed in this way it is evident, that like Jevons himself, our observers (21–40-yr-old) tend to slightly over-count small numbers (numerosity 4–6; *t*>3.567, *df* = 37, *p*<0.001) and undercount the larger numbers (numerosity 8–10; *t*>2.033, *df* = 37, *p*<0.0493). These errors are small, but systematic and significant. We are unsure of the origins of these errors in counting, but Jevons characterized it as “an instance of that inevitable bias in mental experiments against which it is impossible to take complete precautions”. Of relevance to the present study is the finding that the oldest group (61–85-yr-old) actually show significantly greater over-counting than the younger groups (21–40-yr-old) in the range of small numbers from 4 to 6 (2-way RM ANOVA: *F*(188,1) = 5.1776; *p* = 0.026). For clarity, we combined the four younger age groups here for comparison.

### Response Latency

**Figure 4:**
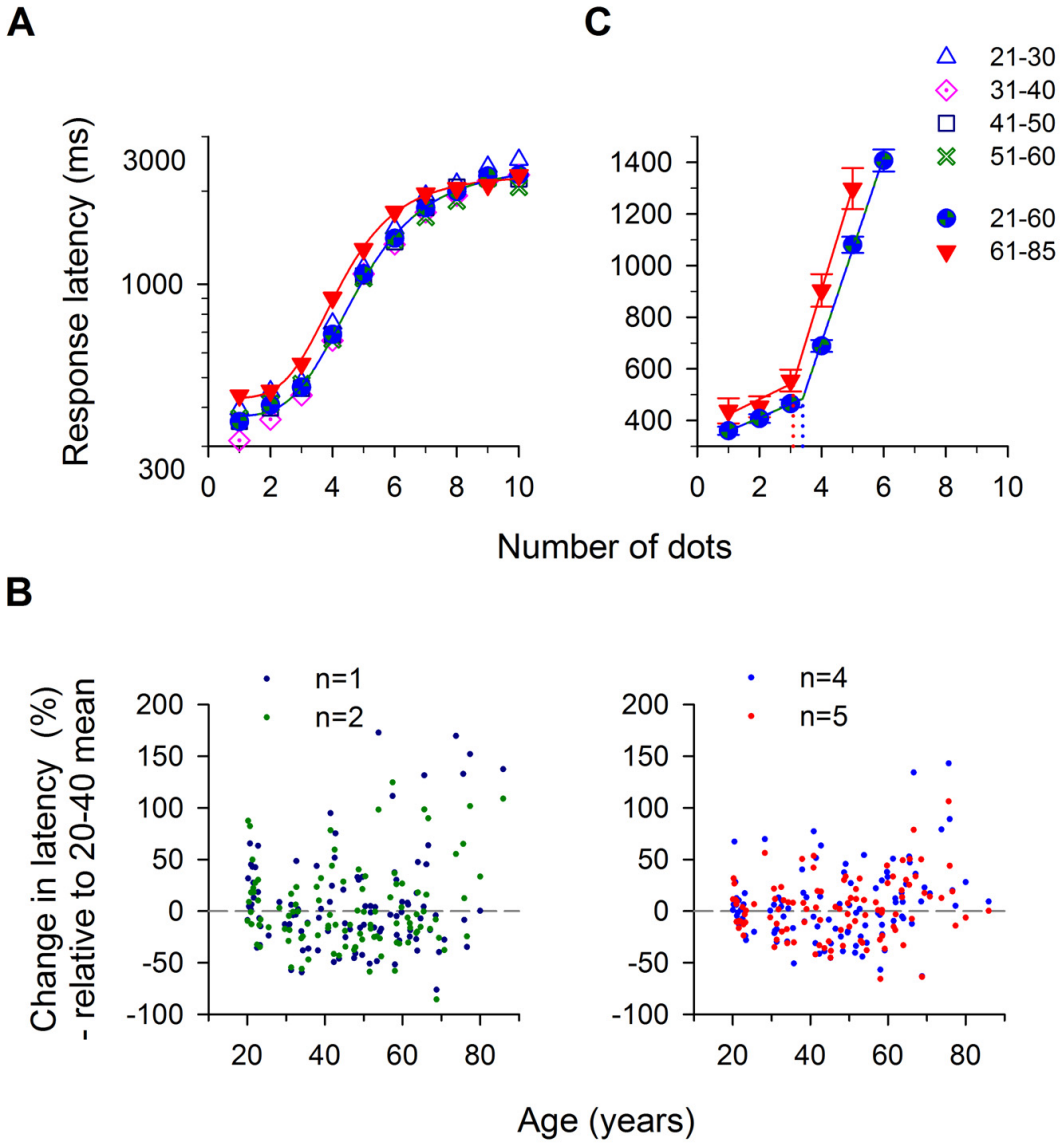
Response latency. (a) Mean response latency as a function of the number of dots. The data of the four younger groups (20–60-yr-old) were combined as shown by a blue curve. For the range of 1 to 6 dots, the latency of the older group is prolonged by 20% when compared with that of the younger group. Note that the symbol legends are listed in panel C. (b) Change in response latency. The latency data is recalculated as percentage change relative to the 20–40-yr-old group mean – positive values indicate longer latencies than the youngest age group, and vice versa. Left panel: numerosity 1 & 2 (subitizing). Right panel: numerosity 4 & 5 (counting). (c) Determination of subitizing span. A bi-linear function was used to fit the mean response latency data, with the intersection point representing the subitizing range. The subitizing speed (the slope before the intersection point) and counting speed (the slope after the intersection point) are both slowed down by 10% in older observers.

The mean latency of the observer's verbal response is plotted on a log scale, as a function of the number of dots in Figure 4A. A logistic function was used to fit the mean latency data. The solid blue and red curves are for observers aged 21–60 and over 60, respectively. We combined the four younger age groups here for comparison. For the age range 21–60-yr, the mean latencies were about 360 to 465 ms for 1 to 3 dots, increased rapidly with increasing dot number and began to saturate at about 2 s with 8 dots. For the age range over 60, the latency curve shifted upward for the range of 1 to 6 dots (response latency prolonged an average of about 20%) and then plateaued to approximately 2 s. The individual latency data are replotted in Figure 4B (left panel, *N* = 1–2; right panel, *N* = 4–5) as percent change relative to the mean data for the age range of 20–40 years, as a function of age.

### Subitizing and Counting

To estimate the range of the subitizing process (subitizing span), similar to most previous studies a bilinear function was used to fit the mean latency data (on a linear scale) versus dot number over the range prior to saturation (i.e., only the first five [older observers] or six [younger observers] data points were used in the regression analysis). In this type of plot, the deflection point defines the subitizing range (Fig. 4C). It is evident that the processing speeds, or slopes, are different before (subitizing: shallower) and after (counting: steeper) the intersection point. For the younger group, the subitizing speed was ≈52 ms/dot and the counting speed was ≈359 ms/dot. The processing speed was prolonged by approximately 10% in the older group (subitizing speed: 58.5 ms/dot; counting speed: 394.7 ms/dot). As indicated by the turning points in the figure, both groups switched from subitizing to counting when there are more than 3 dots. Our data fitting to the mean data revealed a comparable subitizing range for both younger and older adults (dotted lines: 21–60 group, 3.4±0.1 dots; 61–85 group, 3.1±0.2 dots).

### Control Experiments

**Figure 5:**
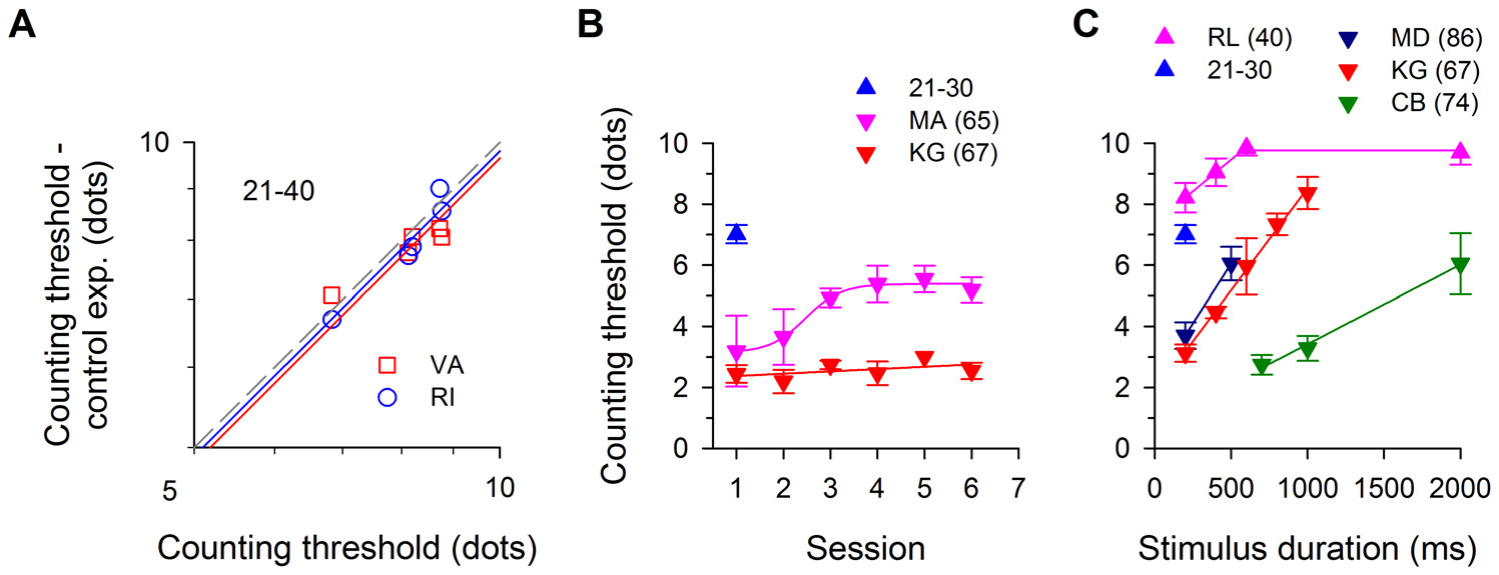
Control experiments. (a) Retinal illumination and optical blurring. The counting performance is minimally affected by lowered retinal illumination (RI, 2%), and optical blurring to 20/20 (VA, 4%) in five young observers. The colored lines represent the average data for these two conditions. A gray, dashed 1∶1 reference line is plotted for comparison. (b) Visual learning. Practice improves counting performance, though to a different extent, in two older adults, however the improved performance is still not comparable with that of young adults. Each training session consisted of 400 trials, a total of 2400 trials in 6 sessions. In this and subsequent panels, observers' age was indicated in parenthesis. (c) Stimulus duration. The effect of stimulus duration is investigated in three older adults with the lowest counting threshold. For comparison, another younger adult was tested for a range of duration.

Our control experiments (Fig. 5A) demonstrate that in five normal young adults (21–40-yr-old), counting threshold was minimally affected by reduced retinal illumination (blue circles) and degraded visual acuity (red squares). The mean threshold decreased very slightly from 8.13±0.35 dots to 7.97±0.39 dots (blue line: decreased by 2%; *paired-t* = 1.336, *p* = 0.2524) and 7.84±0.21 dots (red line: decreased by 4%; *paired-t* = 1.796, *p* = 0.1468) for the lowered stimulus brightness and the optical blurring conditions, respectively (Fig. 5A). Note that the colored lines represent the average data of the five observers, not regression lines.

In another experiment we asked whether practicing the task would improve performance. Two elderly observers (MA: 65- yr-old; KG: 67-yr-old) practiced the counting task repetitively. Each of them had given a total of 2000–2400 responses in 6 training sessions, and showed approximately 20% (KG) and 60% (MA) improvements in threshold (mean of the first two sessions/mean of the last two sessions) across sessions (Fig. 5B). However, the improved performance was still below the mean performance of the 21–30 age group (blue triangle). This shows that, while experience may help, it does not compensate for the effects of age on visual counting.

Another factor affecting counting performance is stimulus duration. Our main interest in this study was on counting in a glance (i.e., with stimuli too brief to allow eye-movements. However, it is interesting to note that with durations greater than 200 ms (where eye-movements may occur), the counting threshold of older adults improves substantially, reaching (and exceeding) young adult levels (blue triangle: 20–30-yr-old group mean - Fig. 5C). In all three older observers, the threshold improved quite linearly with increasing stimulus duration - about 0.67, 0.26 and 0.78 dots per 100 ms for observers KG, CB and MD (67-, 74- & 86-yr-old), respectively, on average 0.57±0.16 dots per 100 ms (or processing time  = 175 ms per dot for the threshold range of 3–6 dots). We wondered how increasing duration affects performance in young adults. Therefore we asked another younger adult to repeat the testing with longer stimulus presentation – his performance improved, again linearly, with longer presentation time and saturated at approximately10 dots at around 600 ms (0.42 dots per 100 ms or processing time  = 241 ms per dot for the threshold range of 8–10 dots). Apparently, this younger observer was able to outperform those older observers at any given duration. Importantly however, over the rising linear range, the processing time per dot does not seem to depend much on age – if anything, the younger observer was a little slower than the older ones.

## Discussion

The present investigation describes how normal aging affects visual counting in a glance. When the stimulus duration is limited to 200 ms, approximately the latency of saccadic eye movements [35], [36], observers do not to have enough time to initiate refixational eye movements to examine each dot in the display. Thus, it is very unlikely that they can make a series of saccades and refixations [22]–[24] to enumerate a large set of dots, beyond the subitizing range.

Our findings provide several new insights into the effect of age on visual counting in a glance. We found that a 30% decrease of the threshold for the oldest age group. Recall that the counting threshold reflects the maximum number of items (out of 10) that an observer can correctly enumerate at a criterion level of performance. Surprisingly, despite decreased threshold, the mean number of dots reported remains veridical– i.e. the ability to count approximately how many visual objects are presented is largely unaffected by age. We refer to this aspect of counting as accuracy, i.e. the mean number of visual objects reported in the present study. Similar observations, but dealing with very large numerosities were reported in a previous study [37]. There, the observers were presented hundreds of dots (*N* = 40–460), and asked to “estimate” the number. The accurate mean counting response with age contrasts with the undercounting that we observe in amblyopes [12], [25]. This suggests that variation in counting performance with age is not a consequence of undercounting.

Most previous aging studies use a key-press to measure response latency [15]–[17], [19], [20], [22], [23]. We were concerned that it may be difficult for elderly adults to make fast hand or finger movements to press a key to indicate their response [21]. In order to obtain a more precise timing measurement, we applied sound detection to measure response time: observers were required to say the number into a microphone as quickly as possible. We also considered and discarded using voice recognition to recognize the number the subjects said, but some numbers could not be recognized perfectly all the times, so we decided to rather have an experimenter to input the observer's response.

An earlier study using a similar voice-key technique to measure voice reaction time to seeing numbers, showed that older subjects can indeed react as fast as younger subjects [22]. Therefore, the delay observed in our older subjects most probably reflects an actual slowdown of enumeration processing, rather than simply age differences in voice reaction time. In general, the response latency we obtained in young adults was considerably shorter (by 150–400 ms) than reported in most other studies [17], [20], [23] in which the measurements were based on a mechanical key-press. One of these studies actually recognized this mechanical delay problem, and conducted control measurements in order to estimate key-press reaction time as an adjustment to their latency findings [22].

Along with visual counting, a wide range of fundamental visual functions deteriorate with age, ranging from low-level (e.g. light detection [38], flicker sensitivity [39], contrast sensitivity [30], Vernier acuity [29] and other hyperacuities [40], [41], visual acuity [32] and contour integration [42]), to high-level (e.g. motion perception [43], biological motion [44], face recognition [45], and stereoacuity [46]). In part, these sensitivity losses are produced by optical changes (deterioration in retinal image quality arising from reduced retinal illumination [47], [48] and increased light scatter [27]) and neural changes (e.g. loss of neurons or decreased processing efficiency at the retinal [49], [50] and cortical [51] levels).

Based on the control experiments, our data clearly show that the loss in counting performance cannot be simply explained on the basis of optical changes. We reduced and equalized retinal illuminance in our younger subjects in order to simulate the optical conditions in the elderly eye, but found no marked decrease in performance. Next, we examined how slight retinal changes affect visual counting. Although our older subjects had 20/20 vision or better, we cannot exclude retinal changes completely as visual acuity gradually decreases with age, by one letter-line from 20/16 to 20/20. Therefore we slightly blurred our younger subjects to 20/20 and evaluated the effect of optical degradation. Again, no marked change in counting performance was observed. The resistance to optical degradation can be attributed to high stimulus visibility (large dot size and high contrast) and wide, very resolvable separation between dots (>20/180).

There is a great deal of evidence showing that the mature brain still retains some amount of plasticity. In a separate experiment, we addressed the question whether the reduced counting performance in older people could be improved to young adult levels. After the practice, our older observers indeed showed some improvements (KG: 20%; MA: 60%). However, their thresholds remain below the mean threshold data of the (unpracticed) younger group. At the very least, these findings support the notion that perceptual learning can improve counting performance, although not fully recover the performance loss, in the aged visual system. Another factor that limits performance is stimulus duration. When given enough time older adults are able to count better, though not as well as younger adults.

Similar to other basic visual functions, it has been suggested that the counting process might represent a primary sensory function, perhaps based on cortical neurons specifically tuned to numerosity processing [9]. From this perspective, our finding of veridical numerical counting accuracy, unchanged by age, would be consistent with the notion that we derive “a statistical description of the scene, where some elements (color, shape, contrast, etc.,) are encoded, together with a rough (±30%) estimate of their numerosity” [8]. On the other hand, the reduction in counting thresholds with age may be more closely related to an attenuation in spatial selective attention [26] or to the ability to retrieve relevant stimulus information, such as contrast and position from visual memory [52] and count the number dot-by-dot, or group-by-group. Psychophysical [53] and neuroimaging [54] data suggest that visual memory remains intact with age. However in those studies, no accurate determination of numerosity was required in performing their spatial-frequency pattern recognition task. It is not clear how the loss of counting performance is related to visual memory and other cognitive skills such as intelligence and information processing [55], but any deterioration of the ability to accurately reconstruct the neural representation of visual scenes could possibly impair counting.

In summary, using a brief stimulus presentation strategy, we provide the evidence that counting accuracy remains unchanged, but the threshold (response variability) and latency increase with advancing age. The loss in threshold might reflect an attenuation in spatial selective attention [26] or visual memory. Our control experiments indicate that the threshold loss cannot be explained by optical, retinal or other perceptual factors, but is cortical in origin. The present findings serve as baseline normative data for measures of counting threshold as a function of age. Future studies are necessary to quantify how distracters with different similarity levels in visual features [23] affect visual counting over the life span.
